# “Cross-border collaboration in onchocerciasis elimination in Uganda: progress, challenges and opportunities from 2008 to 2013”

**DOI:** 10.1186/s12992-018-0333-1

**Published:** 2018-02-06

**Authors:** Thomson Lakwo, Tony Ukety, Didier Bakajika, Edridah Tukahebwa, Pitchouna Awaca, Uche Amazigo

**Affiliations:** 1 Vector Control Division, Ministry of Health, Kampala, Uganda; 2 Retired Consultant Ophthalmologist & NTD Expert, Bunia, Democratic Republic of Congo; 3 Regional Office for West Africa, Sightsavers, Accra, Ghana; 4 Programme National pour la lutte des Maladies Tropicales Négligées et la Chimiothérapie Préventive (MTN/CTP), Kinshasa, Democratic Republic of Congo; 5 Pan-African Community Initiative on Education and Health (PACIEH) and University of Nigeria, Nsukka, Enugu, Nigeria

**Keywords:** Onchocerciasis, Cross-border, Progress, Challenges, Opportunities, DRC, Uganda

## Abstract

**Background:**

Until recently onchocerciasis was prevalent in 37 out of 112 districts of Uganda with at least 3.8 million people at risk of contracting the disease, but following the launching of community-directed treatment with ivermectin (CDTI) in 1996 and the adoption of an onchocerciasis elimination policy in 2007, the country has made significant progress in combating the disease. By 2015, interruption of transmission had been achieved in ten of the 17 onchocerciasis foci, but cross-border foci remained particularly problematic, and therefore within the onchocerciasis elimination framework, Uganda embarked upon addressing these issues with its neighbouring countries, namely the Democratic Republic of Congo (DRC) and South Sudan. This paper summarises the experience of Uganda in addressing cross-border issues on onchocerciasis elimination with DRC.

**Main achievements and lessons learned:**

The key achievements comprise of the adoption of an elimination policy by the Government of Uganda, cross-border meetings, training DRC technical staff and entomological/ epidemiological surveys. The first strategy meeting was held in Kampala in 2008, but the second strategy meeting was not held in Kinshasa until 2013. The involvement of the high-level officials from the Ministry of Health of DRC was critical for the success of the second strategy meeting, and was precipitated by collaboration to control an outbreak of Ebola Virus. Both meetings demonstrated the political commitment of endemic countries and allowed the implementation of a joint action plan. Important steps in establishing a mutually respected elimination targets was agreed on during cross border meetings.

The African Programme for Onchocerciasis Control facilitated and funded these initial meetings, thus overcoming some political and financial challenges faced by both countries. This highlighted the need for multilateral organisations such as the Expanded Special Project for the Elimination of Neglected Tropical Diseases in cross-border activities for other Neglected Tropical Diseases.

The collaboration between both countries facilitated the training of technical staff from DRC in entomology which facilitated joint cross-border activities to update the epidemiological understanding of onchocerciasis in Beni and Mahagi districts in North Kivu and Ituri Provinces respectively. In Nebbi district, Uganda, 23.7% of crabs were infested by the vector *Simulium neavei* compared with 6.3% in Mahagi district, DRC. Rapid Epidemiological Assessment (REA) revealed nodule prevalence of 3.2% and onchodermatitis at 26.4% from five villages in DRC.

**Conclusion:**

Political commitment of both countries and the support from APOC allowed two cross-border meetings which were critical for the implementation of initial cross border activities for onchocerciasis elimination.

## Background

Onchocerciasis or river blindness is one of the Neglected Tropical Diseases (NTDs) recognised by the World Health Organization (WHO). It mostly affects communities close to fast flowing rivers and streams; and has kept people away from some of the most fertile land in Africa [1]. It is caused by infection with a parasitic filarial worm (*Onchocerca volvulus*) which is transmitted by blood-sucking black flies (Diptera: Simuliidae) of the genus *Simulium*. Recent estimates indicate that 37 million people are infected with the onchocerciasis and 187 million people are at risk of infection in countries where the disease is present [2]. In Uganda it is transmitted by members of the *S. damnosum* complex and by *S. neavei*, depending upon the disease focus. Before the advent of onchocerciasis control in Uganda, 37 out of 112 districts excluding Victoria Nile focus were affected by the disease with at least 3.8 million people requiring ivermectin mass drug administration (MDA) [3]. The government started a control programme in the early 1990’s with the support of partners [4], but the good progress made allowed the roll out of an elimination policy in 2007. Since the launching of the policy, 10 of the 17 foci have achieved interruption of onchocerciasis transmission and interventions have been halted in some of them [5–7].

Despite the good progress made in achieving interruption of transmission in a number of foci in Uganda, serious challenges remain with cross-border threats from the Democratic Republic of Congo (DRC) and South Sudan where the disease is also endemic. Lack of effective control in both of these countries could endanger the achievements that have already been made in Uganda by possible migration of parasites with people or vectors into Uganda. The Ministry of Health, Uganda started to address cross border issues in 2008 with support from the World Health Organization African Programme for Onchocerciasis Control (WHO-APOC).

This paper describes the progress, challenges and opportunities in addressing cross-border collaboration between Uganda and DRC in onchocerciasis elimination efforts from 2008 to 2013.

The two Ugandan onchocerciasis foci of interest close to the Uganda-DRC borders are Lhubiriha in western Uganda and Nyagak-Bondo in north-western Uganda. While on the DRC side the areas adjacent to these foci include Beni district (adjacent to Lhubiriha focus) and Mahagi district (adjacent to Nyagak-Bondo focus which includes Nebbi). The two foci in Uganda are each similar to their respective (adjacent) districts in DRC. Beni district is characterized by open plain in the south, and in the north it is occupied mainly by Virunga National Park to the North which is more mountainous with lower temperatures. This is drained by the River Semliki and its tributaries. Mahagi district is typical savannah including some gallery forests along rivers which have sources in extensive swamps. Some of these swamps form the sources of rivers Nyagak and Nyarwodo that extend to Uganda. The population in Beni is mainly subsistence farmers and the common language widely spoken is Rukonjo, and to some extent also Swahili and Lingala. In Mahagi, the population consists mostly of subsistence farmers growing both food and cash crops, and some are involved in cross-border trade between Nebbi and Mahagi towns. The common languages here are Alur, Swahili, Okebu and Lingala. The main rivers are Omi, Nyagak, Nyarwodo and Kakoyi. These two areas in DRC have established functional health systems although the health units are sparsely distributed. The road network is not very good and reaching communities requires trekking on foot in some remote areas.

### Main achievements

#### Cross-border meetings between Uganda and DRC

Two cross-border meetings were held in Kampala (Uganda) and Kinshasa (DRC) in 2008 and 2013 respectively. The first meeting, held on 14–15 August 2008 in Kampala, was organised following the adoption of onchocerciasis elimination policy by the Government of Uganda in 2007. The meeting was attended by 37 representatives from the Uganda Ministry of Health (MoH), the Carter Center, district onchocerciasis teams from Uganda and DRC, one representative from the MoH of DRC in Kinshasa, the chairman of the Uganda Onchocerciasis Elimination Expert Advisory Committee (UOEEAC), the Director of APOC and representatives from WHO Geneva and Uganda. The discussions focused on identifying critical areas for collaboration and one of the key recommendations was to build capacity of technical staff in DRC to support cross-border activities.

The second meeting which was scheduled to take place the following year in DRC was unfortunately delayed for almost 4 years. In the meantime, informal meetings took place as side meetings of the APOC Joint Action Forum (JAF) in Kuwait and Bujumbura or during the sessions of the World Health Assembly (WHA) in Geneva between 2009 and mid-2012. Following the Ebola Virus and Haemorrhagic Fever outbreaks in 2012 in both DRC and Uganda [8], the high-level MoH authority in DRC officially invited (on 18th September 2012) his counterpart in Uganda, through WHO Country Offices in Kinshasa and Kampala, to collaborate and jointly fight the declared Ebola outbreaks across both countries. He also extended this collaboration towards the control of trypanosomiasis and plague, and made a commitment to address onchocerciasis cross-border activities. Therefore, the second onchocerciasis cross-border meeting took place on 18–20 July 2013 in Kinshasa. It was attended by 45 participants from the MoH of DRC at central level as well as from the CDTI projects bordering Uganda, delegates from the Ugandan Ministry of Health, representatives from supporting partners, WHO/DRC and WHO-APOC. Participants reviewed the level of implementation of the recommendations of the first meeting held in 2008 but realized that only two out of seven recommendations had been implemented. A comprehensive action plan was therefore developed for cross-border activities to be undertaken in the eastern part of DRC. However, the plan was developed with the hope that DRC will adopt onchocerciasis elimination policy with time.

Both meetings were coordinated, facilitated and funded by WHO-APOC in collaboration with WHO Headquarters in Geneva and the WHO Country Offices, and the MoH of the respective countries. One thing to be noted was the long delay between the two meetings can mainly be attributed to the slow process of securing financial support to convene the second meeting, bureaucracy in communication through high political offices and the procedures in obtaining invitation letters from both countries.

#### Training of technical staff from DRC

One of the recommendations in the first meeting in 2008 concerned the need for capacity building of technical staff in DRC. Towards that end, four technical staffs were selected from Ituri-Nord and Beni-Butembo community-directed treatment with ivermectin (CDTI) projects in the DRC to receive 1 month of training in Kampala, funded by APOC. The training was both theoretical and practical, and focused on the biology of the parasite, onchocerciasis epidemiology and entomology, field survey techniques, evaluation, data collection, and analysis and preparation of field reports. This training was based on standard WHO training procedures [9, 10]. Following the theoretical training in Kampala, field practical activities were conducted in Beni and Mahagi districts in DRC as well as in Nebbi and Kasese districts in Uganda.

#### Entomological survey

Mapping of vector breeding sites were conducted in all accessible rivers such as Semiliki, Kakoyi, Nyagak, Nyarwodo and Omi based on WHO procedures [9]. Freshwater crabs were caught using locally designed funnel shaped basket traps baited with fresh meat. These were left in rivers for approximately 1 h and later examined for crabs carrying larvae and pupae of *Similium neavei* [9]. Crab collection and examination were conducted in 32 sites during the period of the visit in Beni and Mahagi districts. Some prospections were done in Nebbi and Kasese districts to ascertain the level of infestation.

The results of collection and examination of crabs in DRC and Uganda are shown in Table 1. There was heavy crab infestation in some river systems in Nebbi and Mahagi districts. There was slightly more crab infestation encountered in Nebbi district in Uganda (23.7%) compared to Mahagi district in DRC (6.3%). No crabs were found carrying *S. neavei* in Beni (in DRC), and this corresponds with the cross-border focus (Lhubiriha focus in Uganda) where a member of the *S. damnosum* complex is already known to be the vector.

**Table 1:** Crab collection and examinations in Nebbi, Beni and Mahagi districts in Uganda and DRC

Country	District	No. of sites	Crabs No. caught	No. positive	% pos	95% CI
Uganda	Nebbi	7	283	67	23.7	0.19±0.29
^a^DRC	Beni	9	49	0	0.0	0.00±0.00
DRC	Mahagi	14	240	15	6.25	0.03±0.09
	**Total**	**30**	**572**	**82**	**14.3**	**0.11±0.17**

^a^Democratic Republic of Congo

#### Epidemiological survey

Rapid Epidemiological Assessment (REA) was conducted in a total of five villages in Beni and Mahagi districts in 2009 to establish the magnitude of onchocerciasis in these two districts. Adults 20 years and above who had been resident in the village for at least 10 years were included for examination. This included examination for palpable nodules and onchocercal skin lesions following WHO procedures [10] and the results are shown in Table 2.

**Table 2:** Prevalence of nodules and onchodermatitis in Beni and Mahagi districts in DRC

District	Village	No examined	Nodules			Onchodermatitis		
			positive	% + ve	95% CI	Positive	% + ve	95% CI
Mahagi	Talla	68	0	0.0	0.00±0.00	5	7.4	0.01±0.02
	Ota	25	1	4.0	0.04±0.12	1	4.0	0.04±0.12
Beni	Kalembo	61	2	3.3	0.11±0.08	11	18.0	0.08±0.28
	Masambo	119	4	3.4	0.00±0.07	48	40.3	0.32±0.49
	Rugetshi	72	4	5.6	0.00±0.11	26	36.1	0.25±0.47
	**Total**	**345**	**11**	**3.2**	**0.01±0.05**	**91**	**26.4**	**0.22±031**

All these villages were classified as hypoendemic on the basis of prevalence of palpable nodules (mean nodule prevalence 3.2%) with moderate cases of onchodermatitis varying from 4.0% to 40.3% (mean 26.4%). Rugetshi village had a highest nodule prevalence of 5.6%, while the highest onchodermatitis prevalence was found in Masambo village at 40.3% all in Beni district.

These preliminary results confirmed the presence of onchocerciasis in Beni and Mahagi districts in eastern part of DRC adjacent to the border with Uganda, although at low levels. In Beni district, the freshwater crabs were all negative and this was not surprising because this is an area where transmission is dominated by *Similium damnosum*, in contrast to Mahagi district where *Similium neavei* is predominant [11]. Further epidemiological and entomological surveys need to be undertaken in these areas to provide data relevant for decision making on appropriate interventions.

### Lessons learned

#### Commitment of endemic countries

Political commitment of endemic countries was found to be critical for the success of cross border collaboration, and cross-border meetings held between Uganda and DRC have demonstrated that cross-border collaborations are feasible between endemic countries in Africa. Uganda showed its engagement towards onchocerciasis elimination by adopting an elimination policy in 2007. Consequently, the country changed its intervention strategies from annual CDTI to semi-annual (two times per year) treatment with or without vector control, and as part of this change an independent oversight committee of international and national experts was also formed in Uganda to advise the national elimination programme. Finally, the MoH also developed national guidelines for certification of human onchocerciasis which stressed the need of addressing cross-border issues. There were formal and informal meetings between Uganda and the delegation from the MoH of DRC, but the 2012 Ebola outbreaks in both countries triggered the commitment of the Congolese Government and facilitated the organisation of the 2nd cross-border meeting in Kinshasa in 2013. Regional agreements among endemic countries may speed up the collaboration process as demonstrated in the implementation of onchocerciasis cross-border collaboration in the Mano River Union in West Africa [12].

### Coordination through multilateral organization

Coordination at the multilateral level is critical for inter-country advocacy, facilitation of communication among endemic countries and mobilizing resources. For instance, after the paradigm shift from onchocerciasis control to onchocerciasis elimination in 2008 [13], APOC was an important and effective advocate for cross-border collaboration. It coordinated meetings between many countries which included, Uganda and DRC; Benin and Nigeria, Burundi and DRC, and Congo and DRC, and between the member countries of the Mano River Union. Following the closure of APOC in December 2015 and the launching of the Expanded Special Project for Elimination of Neglected Tropical Diseases (ESPEN) on 23rd May 2016 [14], there is a need for this new initiative to focus on cross-border matters in addition to its main mandate of strengthening national programmes and help secure sustainable financing to combat diseases of poverty. The involvement of ESPEN will certainly speed up and facilitate communication between endemic countries and overcome some political and administrative challenges among sovereign countries.

## Capacity building

Capacity building of technical staff is critical in any cross-border collaboration undertaking in achieving and sustaining collaborative efforts. For example, the DRC-Uganda cross-border collaboration was successful in undertaking capacity building of technical staff based on the recommendations of the first meeting. For example, the training of DRC technicians helped to strengthen the North Ituri CDTI project in DRC, to have health workers capable of undertaking entomological and epidemiological surveys within their local settings, which is a necessity for onchocerciasis elimination on both sides of the border.

## Local level collaboration

Local level collaboration was found to be critical especially in districts which are adjacent across the border. The political leadership across the borders played very critical role in administrative arrangements related to facilitation of teams undertaking surveys, ensuring security and the provision of field-guides. An additional role for local collaboration was the provision of logistics and the supplies necessary for undertaking surveys. It should be noted that in all the surveys conducted the involvement of local leaders (Resident District Commissioners, Chiefs, etc) was critical to the success. For instance, in Beni district in DRC, a chief in one of the villages provided a local map to help in identification of some key rivers and also offered his personal security to escort the team.

## Setting mutually respected elimination targets

Mutually agreed and respected steps in achieving elimination targets is very critical for cross-border collaboration. This will necessitate establishing operational structures at all levels. During the meetings held in the two countries, important steps in achieving elimination targets were outlined and they constituted part of the recommendations. Key steps included:

- Adoption of onchocerciasis elimination policy.
- Development of national Onchocerciasis elimination guidelines.
- Establishment of an oversight committee to advise the programme.
- Identification of strengths, weaknesses and bottlenecks in cross-border operations.
- Adoption of innovative strategies that can accelerate onchocerciasis elimination.
- Regular update of entomological and epidemiological data across-border areas.
- Conducting joint cross-border CDTI activities.
- Targeted supervision of CDTI activities in poor performing communities..
- Strengthening monitoring and evaluations across all borders.

The implementation of these steps by countries was quite problematic due to country specific political, social and economic situations.

## Challenges

Various challenges related to the organization and field implementation of cross-border activities were recorded. A language barrier (Anglophone versus Francophone) slowed the process of communication between participants from two countries at international level. However, at local levels this challenge was overcome because people on the different sides of the border speak the same language. During the training of DRC technicians, a translator in French was hired to facilitate during the theoretical part of the training in Kampala. Additionally, translation equipment was used during all the meetings, yet hiring this equipment was quite expensive and sometimes unavailable.

Secondly, country teams had to cross international borders, thus the need for entry visa. During the cross-border activities in 2009, temporary travel permits were obtained to enter DRC at the international border posts of Mpondwe and Goli in Kasese and Nebbi districts, respectively. However, the permits were not multiple entry and payments had to be made each time the team needed to re-enter DRC. In addition, the permit restricted the movement of the Uganda team to not more than 50 km from the border post. In Beni district, the team was not allowed to go beyond river Semiliki Bridge, while in Mahagi they could not prospect rivers in Djungu district.

Thirdly, logistics and field supplies are obviously critical for cross border activities. A strong 4WD vehicle and equipment like skin snip punches and microscopes are all vital for conducting joint activities. All these vital logistics and supplies were provided by the Uganda National Programme for Onchocerciasis Elimination and crossing the border to DRC with a Ugandan-registered vehicle was quite challenging for the team due to exorbitant tax charges. Some other challenges encountered were the bad road conditions in DRC, and this compelled the team to hire motorcycles for reaching some remote and isolated areas. Therefore, it is imperative that host country takes some ownership of the process and provides logistics and field supplies during joint cross-border activities to avoid these extra and un-necessary expenses.

Finally, availability of operational funds is important for cross-border activities. During the first 5 years of implementation of onchocerciasis elimination programme in Uganda, APOC funded most of the initial steps of cross-border activities with DRC. For sustainability purposes, endemic countries are encouraged to mobilize domestic funding and strengthen collaboration with existing or new partners.

## Opportunities

Relevant opportunities to support and promote the implementation of cross-border activities were reviewed within the framework of NTD elimination in Africa. Stakeholders were consulted during the process of regional meetings on how this agenda could be moved forward [14].

In Uganda and DRC, there are existing partners who have the capacity to promote cross-border activities, namely the Carter Center, Christofel Blinden Mission (CBM), Envision/RTI, Light for the World and Sightsavers. Most of these partners have shown interest to support cross-border activities and countries could take advantage of this available opportunity.

As is already happening in West Africa [15], existing sub-regional bodies such as Economic Community of West African States (ECOWAS), Mano River Union (MRU), Economic Community of Central African States (ECCAS), and Southern African Development Community (SADC) can be utilized to promote cross-border activities in order to fulfil the NTD elimination goal stipulated in WHO NTD Roadmap [16]. Similarly, in the East African region, the East African Community (EAC) platform can be used to move this agenda forward.

## Conclusions

Uganda and DRC collaboration provides some key lessons that can be used to improve future operations in onchocerciasis control and elimination in Africa. Political commitment of both countries enabled policy makers, technical staff and supporting partners to jointly discuss how to address onchocerciasis elimination cross-border activities. The involvement of APOC was critical for the success of these meetings. Further comprehensive entomological and epidemiological studies are required on the DRC side to enable the magnitude of the disease to be established. This would protect Uganda from the resurgence of onchocerciasis in the foci which have already achieved the interruption of transmission. There is need for ESPEN to focus on cross-border issues in addition to its main mandate of strengthening national programmes and help secure sustainable financing to combat diseases of poverty.

## Abbreviations

APOC: the African Programme for Onchocerciasis Control
CBM: Christofel Blinden Mission
CDTI: Community-Directed Treatment with Ivermectin
CI: Confidence intervals
DRC: the Democratic Republic of Congo
EAC: East African Community
ECCAS: Economic Community of Central African States
ECOWAS: Economic Community of West African States
JAF: Joint Action Forum
MDA: Mass Drug Administration
NTDs: Neglected Tropical Diseases
REA: Rapid Epidemiological Assessment
SADC: Southern African Development Community
WHA: World Health Assembly
WHO: the World Health Organization
